# Moral injury: understanding Swedish veterans who are assessed but not diagnosed with PTSD

**DOI:** 10.3389/fpsyt.2023.1200869

**Published:** 2023-12-04

**Authors:** Jan Grimell

**Affiliations:** Department of Sociology, Faculty of Social Sciences, Uppsala University, Uppsala, Sweden

**Keywords:** veteran, deteriorating mental health, suffering, moral injury, identity, ME

## Abstract

This article is based on an interview study of 24 Swedish veterans who experienced deteriorating mental health and increased suffering without meeting the criteria for a PTSD diagnosis. With no clinical answers as to the cause of their deteriorating mental health, they have been thrown into a veteran’s health limbo. The analysis was based on an inductive logic. A key finding of the analysis was a kind of deep-seated permanent moral conflict that could be conceptualized as moral injury. Such an injury can give rise to intense guilt, shame, anxiety, anger, dejection, bitterness, identity issues and more. The results section of the article details five different yet for the sample representative cases of moral injury and their implications. The notion of moral injury is linked to Mead’s division of the self into an *I* and *me*, where *me* is the socially constructed part of the self that is charged with the morality of a group. Thus, a moral *me* played a key role in the development of moral injury. The conceptual apparatus illustrates a new way of understanding experiences that can create suffering and negatively impact a veteran’s mental health. Future research is encouraged that examines this topic, national designs for addressing moral injury, screening for moral injury, and methods for healing included.

## Introduction

Post-traumatic stress disorder (PTSD) has become the diagnosis of our time for veterans who are suffering mental health issues in the aftermath of deployments and operations. This is a relatively new young diagnostic concept that began to be formalized in the 1980s (1). However, the understanding that veterans can change and develop a state of deteriorating mental health during and after deployments is probably as old as organized warfare itself (2). Many of the terms used to describe deteriorating mental health in the 19th and 20th centuries reflected the typical and contextual nature of warfare at the time: nostalgia, soldier’s heart, shell shock, battle fatigue or combat fatigue (1). The development of PTSD has included a diagnostic narrowing and weeding out of previous disease concepts that enriched understanding of the stresses that different operational environments placed on physical and mental health and gave a name to something that cannot easily be captured in diagnostic criteria, but can still be painful and difficult to live with as a veteran. Internal suffering that significantly curtails life does not necessarily fit into a diagnostic manual.

This raises the question of how to understand and think about a deterioration in mental health that does not lead to clinical PTSD. Since the mid-1990s, there has been a burgeoning and growing field of social science and humanities research that has developed interesting new concepts that do not have a medical or clinical status. These concepts are important because they shine a spotlight on both gaps in a clinical conceptual apparatus and the complexity of a deterioration in mental health that is not necessarily pathological. One such example is moral injury. Moral injury as a concept has the capacity to nuance the complexity of deteriorating mental health, with or without PTSD (3–5). Moral injury can be explained in simple terms as a type of deep-rooted, permanent moral conflict that can create guilt, anxiety, anger, frustration, bitterness, depression, identity issues and more (6–10). A moral injury can occur when deeply held moral beliefs (11) or identities (12) are betrayed or transgressed by the individual or others. An individual may have different roles within such an event(s) or process(es), for instance, as an actor/perpetrator, witness, and/or victim. However, betrayal of what is considered morally right (13) cannot be approached in universal terms; there is a highly subjective dimension to moral injury. One person may respond to an incident in morally conflicting ways which if left unresolved may lead into a deep-seated moral injury. Another person may react to the same incident in other less morally encumbered ways.

The concept of moral injury can provide insights to veterans who feel unwell yet do not meet diagnostic criteria for PTSD, which is a challenge for both the individual and their families. Such an undiagnosed deterioration in mental health is illustrated at the National Veterans Clinic at Uppsala University Hospital in Sweden.^1^ Half of the patient flow in the clinic does not fulfill the criteria for PTSD despite experiencing a marked deterioration in mental health and increased suffering (14). For some of these patients, an exploration of moral injury can bring clarity to their sufferings. In addition, both the individual and family members are given a conceptual name for the condition that they can use to help them understand the situation and seek possible paths to healing.

This article investigates in what ways a deterioration in mental health can manifest as moral injury among deployed veterans/patients from the Veterans Clinic. From the sample of 24 interviewees who described various types of moral concerns and conflicts, five representative cases have been selected in order to detail different manifestations of moral injury and their implications for increased suffering.

### Research on moral injury syndrome and recovery in veterans

Contemporary research on moral injury in relation to mental health issues among veterans started in a clinical veteran context with Shay and Munroe’s (13) ground-breaking work on betrayal based moral injury in the 1990s. They found, among other things, that Vietnam veterans with PTSD also articulated a kind of perceived betrayal of what was considered morally right, a conflict for which traditional therapies did not work well [also see (4, 5)]. Therefore, other types of unconventional methods were developed to therapeutically support recovery and pain relief. Decades later additional research has confirmed and continued to unfold the co-occurrence of moral injury and PTSD among veterans with mental health issues (3, 15, 16).

Yet more than a decade elapsed before a seminal article by Litz et al. (11) gained a wider and interdisciplinary interest and audience and thereby accelerated research on moral injury, which will be detailed later. In addition to stimulating researchers from various disciplines, this second wave development started to gain a firm foothold among soldiers and within veteran communities as well. Litz et al. (11) argued that moral injury can occur if an individual fails to prevent, witnesses, or gains knowledge of actions that violate deeply held moral values and expectations of the self. Such injury is subjective and process-related, and may involve actions, events, or knowledge that may not initially be perceived as morally problematic but that sometime later may be perceived as violations of a personal moral compass. This understanding also creates different roles that illustrate the complexity of moral injury. For example, a perpetrator whose actions violate a moral compass may experience guilt as a dominant problem complex, as may a witness to an event who failed to take action. The complexity is further compounded by the fact that a person may experience multiple roles, such as perpetrator, witness and victim, at different stages of an event or due to multiple types of events. The role perspective points to the third and most recent conceptual development.

The third wave development of moral injury has a distinct link to the concept of identity (3, 12). A moral injury is understood in light of morally charged identities or a moral *me*. A certain moral charge of identities sprung from different group contexts is necessary for the self to participate in communities and to navigate life’s various group affiliations with a relatively intact and unharmed conscience. If a person violates and abandons the morally right, there is a risk that a morally charged identity will clash, become severely injured and can no longer be credibly sustained, hence moral injury occurs and is understood as a failing of identity or character.

Also, research how to best and most effectively treat moral injury has intensified in recent years as the conceptualization, qualitative and quantitative research, and the establishment of the concept have advanced significantly. The research body on recovery from moral injury can be said to consist of two sub-orientations: one emanates from a clinical context [e.g., (11, 17); see ongoing clinical trials with Acceptance and Commitment Therapy for moral injury by Borges (18)] and the other from a context of pastoral and spiritual care and counseling and religious traditions of wisdom around spiritual healing [e.g., (8, 16, 19–21)].

### Moral injury as a construct

While empirical studies contribute to evidence for understanding the scope, dimensions, and implications of moral injury [e.g., (22–27)], theoretical work attempts to clarify its definition [e.g., (3, 7, 10, 12, 28)] so it can be developed and refined as an operationalized construct [e.g., (29–33)].

The operational definition of moral injury in this article is an experience that emanates from an incident, situation, or episode in life, which with time grows into an emotional and thought complex about right and wrong tailored to various narrative positions (e.g., actor, witness, victim). The emotional complex includes, but is not limited to, storied manifestations of anger, bitterness, sadness, depressiveness, as well as, feelings of regret and anxiety. The thought complex can involve ruminations of perceived betrayal, transgression, guilt, and/or shame tailored to narrative positions. The difference between moral distress/conflict and moral injury is that distress/conflict is potentially within the confines of problem solving. A moral injury has passed this stage and is therefore a permanent painful emotional and thought structure in the self.

A moral injury can lead to intense experiences of guilt, shame, disgust, bitterness, anger, depression, anxiety, regret, failure, decreased zest for life, lack of joy in life and identity issues (7, 10, 11, 28, 34–36). These problem complexes are difficult to handle and may co-exist with clinical PTSD or occur independently of clinically diagnosed PTSD (3, 15). When moral injury is comorbid with PTSD there is increased for suicidal ideation and suicide attempts (37), yet the two appear distinct: While PTSD can be described as a biologically based disruption of the threat-response system, the absence of strong connections between moral injury and symptoms such as hyperarousal and reexperiencing suggests that for now moral injury is better defined by moral injury events and their sequelae (3). Thus some researchers suggest that moral injury may be mediated by pathways distinct from threat-based traumatic illness. Also, individuals’ appraisals of the morally injurious experience(s) may be accurate and even desired by societies (as to keep human behaviors morally desirable), and thus cognitive restructuring practices may be inutile or possibly even harmful (12, 38). To feel guilty when transgressing deeply held moral beliefs is not pathological (26). Shame and guilt (the primary emotional reactions of moral injury) are fundamentally different than fear and anxiety (the primary emotional reactions of PTSD), which may mean that exposure techniques used during PTSD therapy are less effective for moral injury (15, 38).

While no clear consensus has yet emerged in defining the construct, there is common ground that is generally agreed upon (9). Moral injury is understood as a dimensional problem that does not have a fixed criterion threshold that must be met for the injury to occur (35, 39). A potential moral injury event does not necessarily lead to a moral injury for the person exposed to the event, since people’s identities are morally charged in different ways and people have varying degrees of resilience and varying internal and external resources to help them face and cope with events. A moral injury is based on a person’s moral and interpretive subjectivity, filtered through moral lenses or identities [(11, 12, 26, 32, 34)]. For some people, this creates internal clashes; for others, there is little or no friction. It is therefore difficult or impossible to say that a traumatic event will lead to a moral injury (27). However, a potential moral injury experience that leads to moral conflicts that are not dealt with may over time grow into a moral injury. This has been illustrated in the integrated moral distress and injury model by Grimell and Nilsson (35).

### Moral conflict within the dialogical self

Social psychological research on identity and morality suggests that identity processes and framing of situations as moral are significantly associated with moral action and moral emotions of guilt and shame which can contribute to mental health problems (40, 41). The presuppositions for a moral injury to develop (i.e., morals, values, morally charged identities) arise in an intimate interaction between the individual and the larger society, institutions as well as the smaller parts of a society (e.g., groups, family and friends). This process includes both objective aspects of morality that are socialized from outside into a human being and a level of subjectivity/individuality due to socialization within smaller groups and interpersonal contexts.

Mead (42) argued that *me* is the part of the self that is developed and created in a social context. *Me* is the part of the self that helps the self to understand, interact and define the situation in the light of the cultural symbols, in particular language, morals, values, meanings and practices, that constitute a me emerging from a particular social context [(42), p. 209]. At the same time, there is another part of the self, the *I*, which is active and acting and which is in the present [(42), p. 77]. The ephemeral part and agent of the self is the *I*, while the *me* is the social roles or cultural characters that society reproduces and creates through the generalized other, which roughly speaking is the society of the self. The morals and values of society take place in the self through a *me*, and each group a person belongs to creates a particular me in the self. Humans therefore have many *mes* to consider that coexist and compete in the self. By means of the morals and values of society and groups, the *me* exercises social governance and control over the *I* [(42), p. 210]. Each group that a person is part of projects its own moral *me* onto the person, which means that a person has many *mes* to consider.

The social and relational perspective is key to understanding the human fabric embedded in moral injury. All morality is social and relational and arises from interactions between people within groups in a society (42). Morality is expressed in the values, beliefs and practices that constitute a group, large or small (40, 41). The generalized other (e.g., the group and its morality) varies in importance and strength in a person’s *me*. The moral impact of the group simply does not take the same form for all people in a group. The empirical container of morality can be said to be an *me*-identity such as veteran, father, mother, husband, wife, teacher, police officer, nurse and so on. Morality becomes observable and visible in identities that one both *is* and *does* in lived life (14, 26). It is in the relationships and actions of lived practice that moral conflicts and injuries can occur through the betrayal of what is perceived as morally right (4, 5), the violation of internal moral compasses or a moral *me* (11), and the failure to maintain one or more morally charged identities (12, 26). Not infrequently, several of these approaches may interact and overlap during an event in which a person may hold positions as perpetrator, witness and victim, or due to events that occur independently of each other and involve different moral dimensions (3). This brings to the fore a more recent understanding of the self that is underpinned by Mead’s thoughts, namely the dialogical self (43–45). The self is extended to society, institutions, groups, family, and friends through various moral mes which mean that *I* need to consider divergence and friction between *mes*. Characteristic of the dialogical self is the value-laden positional context of the self that *I* have to constantly navigate. Dialog is a distinguishing feature of the self but not easily achieved due to conflicting moral *mes*. In the analysis, *me* equates to narrative identity, which in a methodological sense is understood as an empirically discernible part of the self, for instance, a salient veteran identity that has emerged in a particular cultural context, with a language, morality, meanings and practices that derive from it (26, 46–48).

An important perspective for understanding why moral injury may occur among veterans in particular is the strong moral charge that *me* has in such groups. For many veterans, a strongly morally charged *me* emerges during service and international deployment that embraces obedience, loyalty, task focus, discipline, community, camaraderie, renunciation of what stems from life at home, and self-sacrifice of one’s own life and the lives of others if things get really bad (49). A strong *me* is needed to be able to perform extreme tasks that are taboo (i.e., harm, kill) or morally transgressive in a peacetime society when the circumstances are life-threatening (50–55). A radicalized veteran identity (26), or warrior mask (56), is so robust that it can temporarily or recurrently put other important identities on hold and ultimately, consciously and unconsciously, sacrifice them on the altar of duty. The realization of the sacrifices made can be painful and difficult to live with (57). In the case of moral injury, identities and events have clashed profoundly, shattering the story of who I am fundamentally. A storied moral injury may include, but is not limited to, feelings such as guilt, shame, disgust, anger, bitterness, sadness, and grief.

## Method

A qualitative interview method was considered particularly appropriate for this study on moral injury due to the lack of qualitative information from primary sources/veterans (58). The study was understanding-driven with a specific intent to uncover the role and topic of moral conflict and injury among the interviewees.

### Characteristics of the Swedish veteran context

A total of 67,093 people have served on international military deployments since 1953, with 54,218 of these veterans still alive as of September 2021 (59). Of the current living population of veterans, 50,631 are men and 3,587 are women. This means that the overseas military context is clearly dominated by men, although it is not an entirely single-sex context. It can therefore be said that the social interaction is based on the interaction between men, and that there is a high degree of homosociality (49, 60). It was not until 1989 that women were allowed to work in the armed forces on equal terms with men, and the Swedish Armed Forces is still one of the most gender-segregated workplaces in Swedish society (61).

Swedish military veterans have typically been serving on short term contracts including the time for the actual mission (about 6 months). Upon the return they have transitioned into civilian life more or less directly. This have left little room for processing and addressing deployment experiences and veteran identity. Systematic support has gradually developed during the past decades yet do not address moral injury in its current form.

When it comes to the number of expatriate veterans deployed from other Swedish agencies, it is more difficult to get a quantitative holistic view of how many there are over time in a Swedish context. However, it can be noted that there is a larger number of deploying agencies in Sweden (e.g., the Red Cross, the Ministry for Foreign Affairs, the Swedish Civil Contingencies Agency, the Police, the Coast Guard, Swedish International Development Cooperation Agency and many more) that send employees to perform duties in an international context. These may be short assignments, but are sometimes much longer placements.

### Sample

Purposeful sampling was used (62, 63). Sampling was done with the support of the Veterans Clinic at Uppsala University Hospital, and 24 veterans were interviewed in the spring of 2022. The participants had been screened for PTSD but were not diagnosed. The medical staff made their selection of patients/participants on following selection criteria: deteriorating mental health and increased suffering related to PTSD symptoms, moral issues, existential issues, and identity. Patients with a clinical diagnose were excluded.

### Procedure

The interviews were conducted during an intense wave of Corona infection in Sweden in early 2022. The interviews were therefore conducted via videoconference or by mobile phone due to a poor internet connection, technical problems or a lack of digital technology that enabled interaction via videoconferencing. One interview was also conducted in a traditional way together in a physical room at the request of the interviewee (once the pandemic subsided in May 2022). Several previous studies conducted via videoconferencing during the pandemic have shown that such interviews work well and are just as informative as the traditional interview method (26, 64).

The interviews were based on a semi-structured interview design which was formalized in an interview guide containing 25 themed interview questions that addressed a number of sub-questions regarding areas such as deteriorating mental health and moral conflicts and injuries. The questions were broad, open-ended and descriptive in nature with no simple answers. For example, the interview questions were formulated as follows.

- If you reflect for a moment on your service/deployment, can you describe the parts or periods that you feel have affected your mental health?
- Are there any events that have had a negative/troublesome/destructive impact on your moral thoughts that you would like to share?
- Would you like to describe how you are feeling today and what has been the path/journey to get there?

The purpose of open-ended interview questions was to encourage interviewees to tell their own story and share the experiences they had from the life they have lived. The open-ended question methodology creates a good opportunity for the interviewee to give their own interview answers (65–68).

The total interview time was 29 h and 30 min.

### Analysis

The planned analysis was an inductive content analysis (58) with a focus on narrative identity claims (26, 46, 48, 69).

## Results

The sample included veterans from both the Swedish Armed Forces and other deploying agencies. Nineteen interviewees were from the Swedish Armed Forces (16 men and 3 women) and five (4 women and 1 man) were deployed by other agencies. A total of 17 men and 7 women were included. To hinder backtracking and to protect the anonymity of the interviewees, the agencies involved have not been specified, apart from the Swedish Armed Forces.

**Figure 1:**
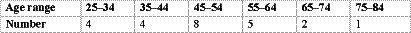
Age distribution and number of participants.

The age distribution of the interviewees was broad and is illustrated below (Figure 1).

The Army, Navy and Air Force, the traditional branches of defense, are represented among the interviewees, although the Army and Navy form the bulk of the military participants. The interviewees illustrate a broad mix of different units from the branches of the armed forces.

The number of overseas deployments varies widely among interviewees. Some have completed 1–2 overseas deployments, while others have completed 3–8 overseas deployments. There are also interviewees who interrupted planned or ongoing deployments for various reasons. The overseas deployments were carried out under different deployment paradigms ranging from the Middle East (e.g., Cyprus, Sinai, Lebanon), the former Yugoslavia (e.g., Macedonia, Bosnia, Kosovo), Africa (e.g., Liberia, Mali), to Afghanistan. However, the mission focus among interviewees can be said to be centered around deployments to the Middle East and the former Yugoslavia in the 1990s as well as deployments to Afghanistan and Mali in the 2010s.

The interviewees from other agencies stem from several major Swedish agencies that sent the interviewees to trouble spots and disaster-stricken areas around the world. These interviewees carried out a large number of qualified overseas operations and missions, and for many, but not all, this mission paradigm is still ongoing. The mission period spans from the 1980s until recently. More broadly described at the group level, the interviewees’ mission breadth has been in the areas of law and order, security, aid, health and migration. In addition, some of these interviewees have some degree of military training and background.

### Analysis

The interview transcriptions were coded in a qualitative analysis software called Atlas.ti. An inductive logic was used during the analysis process, which involved a movement from many individual small codes to general overall themes. The process was based on coding important and interesting individual findings in the interview material and then developing the observations into general themes or group-level categories called code families in Atlas.ti. An inductive logic starts from the experiences of the individual participant and is later organized at the group level. New knowledge, hypotheses and theories can be constructed and developed using the information at the group level (58).

Thirteen code families accommodated a larger number of individual codes. Not all individual codes are automatically reflected in a code family. However, the associations of a code should lead to the code family and vice versa. Some of the individual nuance is lost in the movement from the individual to the general. At the same time, in an inductive process, individual coding needs to be sorted in some kind of way in order to create overview and order, otherwise it becomes difficult to describe the results of the analysis in a meaningful way. The following 13 code families organized all the individual codes.

1. Code family:Military and veteran identity.
2. Code family:Military culture/mission culture.
3. Code family:Missions and experiences.
4. Code family:Homecoming/transition experiences.
5. Code family:Peace society and the gap to civilians.
6. Code family:Approaching deteriorating mental health in relation to the Self and family.
7. Code family:Different symptoms of mental illness and PTSD.
8. Code family: Moral conflicts and injuries.
9. Code family: Existential doubts and life issues/views.
10. Code family:Alcohol and cannabis to reduce symptoms and anxiety.
11. Code family:Primary care, Veterans Clinic, medical support.
12. Code family:PTG (post-traumatic growth), growth and strategies.
13. Code family:veteran community and camaraderie.

Code family 8 was found to be a particularly significant category in order to understand deterioration in mental health that does not lead to clinical PTSD. All code families have been published elsewhere in a Swedish book (14).

In order to take the analysis to an even higher level, an abductive approach has been applied where analysis and concepts were allowed to cross-pollinate in the results section.

### Selection of cases

All interviewees in the study struggled to varying degrees with different types of moral conflicts and injuries that were directly or indirectly related to the deployments. Moral injury is considered a key dimension in understanding the interviewees’ deteriorating mental health and increased suffering. The different developmental waves of moral injury were illustrated among the participants, but could also be combined in a single participant. However, all 24 interviewees cannot be presented in a single article which intends to flesh out the moral topic in detail.

In the results section, a number of representative yet dissimilar cases will be elaborated that illustrate the breadth of moral injury found among the interview participants. Each of these moral injuries recurs in different forms, with varying intensity, among the other participants in the study. The cases in the result section have been carefully selected to speak for the entire sample yet ensure a wide range of ages, genders, deploying agencies, and types of service and missions. The cases selected present how the moral injury occurred, what kind of suffering the injuries have caused, different time perspectives, and the challenges of healing. The five cases provide a unique insight into how lives, selves and identities have been affected by various moral injuries. Such exhaustive qualitative research in the continuing efforts of nuancing the understanding of moral injury.

In the presentation of the results, grades, positions, specific deployment areas and other detailed information, including age, have been omitted to hinder backtracking. Each interviewee was asked to create their own fictitious name.

#### To suffer from a 30-year-old moral injury: Harald’s case

Harald had carried out many international deployments and missions for deploying agencies other than the Swedish Armed Forces. He was in the 55–64 age range. Harald had a well-established family and professional life. His first deployment was in the early 1990s and his last one was completed in the 2000s. Harald lived with a lifelong moral injury, where pain and guilt were pronounced. During an international mission in a conflict-affected area, Harald had been given a specific assignment to manage, for which he enlisted the help of a number of local people. After some time, these people began to disappear. Harald realized that they had been taken into custody by an entity of that country and did everything he could to get them released. But Harald was alone and without real organizational resources and support. The whole situation was, of course, distressing and difficult. After some time, several of the employees were found dead. Harald felt bad about it, but completed the mission and then went home. The operation had not been entirely negative, Harald had succeeded in the mission. But the incident left a deep mark on him for the rest of his life.

This is how Harald described it:

Nothing has impacted me as much as this has. I feel an enormous guilt for these people who took … I feel … I was convinced for an incredibly long time that it was my fault. I had recruited these people and that’s why they died. And I got help so I’ve managed to shake off that guilt. But I still feel a great sadness when I talk about it. And I’ve been reminded of this in different ways, as naturally happens when you are in situations where there are weapons and there’s violence and there are vulnerable people who are dying or at risk of dying. Then I also carry this with me. But there is nothing that compares to this situation.

For Harald, the way he felt in the life that followed had gone up and down based on the incident, and he described it like this:

It's been heavy at times. I have periods where I think I've managed to push it down and have been able to live with it for quite long periods without it bubbling up [It bubbled to the surface again when Harald started a family and developed identities as a husband and a dad]. And there's a lot related to this guilt that's come out in the last few years. All of a sudden it's become so incredibly heavy to bear, and I've talked a lot about guilt and responsibility and got help to look at things differently. That there were actually other people who put you in this situation. That there were higher-ups who had access to information. There were those who had better access to analysis, who should have been able to predict this turn of events. So, I've gotten help through therapy to enable me to look at it differently. But at times I've thought very strongly that I had a very direct responsibility for these people's lives. […] It's affected my thinking, it's affected my … I think I've isolated myself socially to some degree at times.

Moral responsibility and moral guilt weighed on Harald, and it affected his mental health and social life. Harald has received therapeutic help in relation to how he thinks about the situation, and things have improved considerably. But it had not been possible to completely resolve the moral guilt issue through cognitive and logical strategies.

Harald said:

My fault. I failed. That's what I've been living with for decades. And it's a moral debt. But it's much better now. Even though I'm still very emotionally affected, cognitively I've been helped to think in a different way and that makes me realize that there were many others who are even more to blame for this than I was, of course.

When asked how the moral guilt felt, Harald replied:

The guilt feels numbing. It's a kind of despair. It's a physical feeling. Especially when the feelings become incredibly strong, of course, as all strong emotions can be felt physically in the body. But then it leads to a lot of thoughts that can be distressing. Existential thoughts. It's something like that.

Harald lived a fulfilling life with family and children and was well established in his field of work, but at the same time he described himself as “a broken man.” He had lived with a demanding moral injury for much of his adult life, but he had buried it behind the secrets of the war machine. His family did not know about the wound he carried and it was something he could not imagine telling them. The moral injury was complex too because Harald had multiple roles during the course of events that can be understood in terms of a betrayal of what was considered morally right (4, 5, 13) and a failure to maintain a morally charged identity (12) given that Harald could not save the people he employed. Harald had a role as a responsible actor because he in a sense set the ball rolling when he hired local people. At the same time, he was a witness who had to watch the event from the sidelines without being able to control or influence the situation. At the same time, he was a victim because he lacked the organizational support and resources needed to resolve the situation that arose. The deploying organization therefore betrayed him in some sense. Harald’s moral injury also highlighted the challenge that research in this field faces today because, given the etiology of the injury, any treatment extends beyond the traditional psychiatric, psychological and therapeutic paradigm (3, 9, 16, 26).

#### To suffer half a life time from military and civilian moral injuries: Fanny’s case

Fanny was in the 65–74 age range. She had completed an international military deployment in the 1990s in a special military position. The deployment was well entrenched in the family. At the time, Fanny had several strong and meaningful identities as a partner, parent and employee, to name a few. The deployment was in many ways demanding, stimulating and eventful. Fanny quickly settled into the position and was entrusted with important tasks, duties and responsibilities. Due to various circumstances and loyalties, Fanny essentially remained in the area of operations for the duration of the deployment. Fanny’s interview narrative is complex from a moral perspective, but two different types of moral injury could be discerned.

The first injury is slightly rewritten here to hinder backtracking and is related to the deployment and her return home. Fanny had some military training but was brought into a vacant position that exceeded her military training background. This was possible because she had civilian skills that were considered equivalent. Fanny quickly settled into the position, was given an increasing range of responsibilities, was entrusted with handling tasks that extended beyond the position, and handled the tasks well. Given her civilian work, she had a capacity that far exceeded that of the military position. However, on her return home, she could not receive a service rating for the position in question because she did not meet the formal military requirements. Fanny was left alone to deal with and make sense of a strong *veteran identity* (46, 70), *the deployment* itself, *the sacrifices* she had made (60), *the return home* (5, 71, 72) and the demoralizing message that her service did not count in a formal military sense. She was never formally seen or acknowledged for the contribution she had made. After this decision, she decided never to tell anyone that she was a veteran again; everything was hidden behind the impenetrable shell of the warrior mask. The incident can be understood as a textbook example of a perceived betrayal by a superior with legitimate power, in relation to what is perceived as the morally right thing to do, in a high-risk situation (4, 5, 13). Outside the military context and on her own, Fanny tried to deal with the moral pain and injury incurred through stoic silence while experiencing the expected and normal transitional challenges. This was an overwhelming task. The downward spiral could not be broken, but instead created an accelerating deterioration in her mental health. In the wake of her life collapsing for a period of time, the next moral injury occurred.

The deterioration in her mental health alienated Fanny from life. Her marriage fell apart, her home was sold, her family was torn apart, and she lost her job. But the children had a special place in Fanny’s suffering, because in the process another moral injury occurred which unfolds after the interview excerpt.

Here is what Fanny had to say:

I have to say, I feel so guilty when it comes to my children. Because it wasn't long ago that they said “Mum, we've always thought that you've acted so strange since you've come home. But we've never been able to tell you that. And we've missed you so much”. And they were the ones who said “We'll go with you to the Veterans Clinic. We'd do anything for you”. […] But I, how could I do that (crying)? Leave them [to deploy]. I just can't understand that. How could I? That's what hurts me so much. […] But I just felt well, no, I'm not bitter (crying), but I don't know how to reconcile that. I feel guilty that I left my children, I really do.

The moral injury in this case can be understood as a perceived failure to maintain a morally charged *me* which equates to an identity as a mother (14). Such an identity is charged with morality partly from societal culture and partly with family-specific morality that indicates what behavior is morally desirable, right and proper for such a character. In addition, there are strong emotions associated with children. For Fanny, her identity as a mother was very clear before the deployment, but in the aftermath of her deteriorating mental health after her return home, Fanny could no longer maintain it in the same way as before. Fanny’s deteriorating mental health can also be understood in the wake of a character breakdown that meant she felt she was betraying her children, which was emotionally charged. The big existential question is how a person, Fanny in this case, could reconcile such a perceived betrayal of the morally right? In Fanny’s case, moral injury can provide an interpretive framework for understanding her lifelong pain and suffering in which she was not only an actor in the act of leaving and a victim of an institutional betrayal, but also a witness to an event that she herself could not prevent (7, 12, 26).

#### To suffer from moral injury due to a moral transgression and gender: Sofia’s case

Sofia, a specialist officer in the 25–34 age range, was on sick leave at the time of the interview. Early in the process of military socialization and identity development, she had suppressed things that she had initially reacted to. Here there was a moral conflict between two contrasting identities and moral outlooks. During military identity formation, this became moral stress and conflict in the self.

During the interview she described it all as follows:

So I applied there [Swedish Armed Forces] for that reason [i.e. it was a physically demanding and practical profession], but I hadn't really thought through what it entailed. And now that I've reflected on it, I've realized that a lot of my values do not align with my professional role. From the first time I held an assault rifle, I found it uncomfortable. […] Something in me … It was very much against my values to be able to hurt another human being. Very early on, there was a very high level of stress in me. There was an internal conflict that I wasn't aware of at the time, which I can see now has affected me a lot. The fact that I was always in some kind of weapons handling, weapons training, when we were at the shooting range, when there was combat training and stuff like that – it never felt good. There was always an internal resistance and a conflict in me because in the end, all of that leads to the fact that we were to train to be as good as possible at killing other people. And that's something I don't want to do.

The Military *me* provided a new context in the self that the I could react to and there was conflict. The friction between a socially created Military *me* was difficult because the *I* reacted with resistance to the social control that the *me* exerted on the *I*. In addition, there was the other Civilian *me*, the moral one consisting of generalized others from a civil society that did not want to harm other people. But the social control of the Military *me* was so strong that the *I* and the other Civilian *me* had to submit to being controlled and subordinated.

Sofia described the process as follows:

I would say that is probably what I call my authentic self. […] And for many, many years I have not listened to that voice. And I think sometime during my basic training when this voice was probably saying something to me, but I wasn't listening. Eventually it shuts down. You lose touch with yourself. And what's left then is the intellect, or the brain as it were. I also lost touch with my feelings, so I've tried to manage my life in other ways. By exercising a lot, working a lot, performing, always being around other people, not being by myself so much. A lot of these kinds of anxiety-reducing behaviors.

Sofia’s moral injury could be said to consist of a violation (11) of an internal moral compass (me) against the background of the military socialization process, which led to the silencing of Sofia’s authentic moral *me* and identity in the self (12, 26). She lost contact with her Civilian *me* and her inner voice. Her awareness of the moral conflict accelerated markedly during the international deployment Sofia undertook in a high-risk military area in the latter part of the 2010s. At that time, she realized that there was an imminent risk of having to use a weapon against another human being. This was reinforced on her return home when she was advised to attend a combat-oriented instructor training course. The identity conflict accelerated to levels that resulted in sick leave and an intense desire to leave the officer profession. This can be understood as a powerful protest from the *I* due to the social control exerted by the Military *me*.

At the same time, Sofia had also experienced poor treatment as a young female officer at the unit at which she was employed while being ambitious and working hard at an officer job that had “no limit” and would never “be done.” She compensated by working harder, exercising harder and performing. There was also a moral betrayal here (4, 5) that gravitated toward the fact that when Sofia was at her most vulnerable as a young newly-graduated officer and needed help and support the most, she was met with anything but help and support.

Sofia described leadership and the working environment as follows:

When I started as an officer, I was treated very poorly. I went in as a young, high-achieving girl, new to the unit and I went into a slightly higher position than you normally do and stepped on a lot of people's toes because of this. So I was subjected to quite a lot of master suppression techniques, bullshit. Yeah, I would call it bullying. I really felt like an outsider. It was very much a case of “You have to prove that you're good enough. Until then, you're worthless”. When I asked for help and tried to be humble and so on, if I was given a task that I felt I hadn't mastered and asked for help, then I got the response “No, you have to figure it out yourself”. There was very harsh jargon from the beginning, which I never liked. My whole first six months I thought about quitting like every day, because I felt it was a real blow to my mental health.

However, Sofia was a hard worker. She eventually settled into her professional role and was able to show what she was made of. She then became one of the gang, affirmed and accepted. However, the problems with the working environment and poor leadership did not end; they continued. The military identity she developed on the military stage became a warrior mask with which she stoically endured the situation while concealing, denying and displacing the suffering and pain she was experiencing (56). Summing up her feelings and thoughts about the whole thing, Sofia said:

I felt incredibly mistreated. There was a lot of bullshit talk and I feel that many of my superiors have been very irresponsible. They haven't looked after me either, but have just been pushing me harder and harder. Yeah, I felt a lot of frustration and anger but also disappointment. […] I felt really angry at people who were directly responsible for the poor treatment I received, but also because the system is set up the way it is.

Given Sofia’s experiences, a military identity can be said to be encoded in accordance with a male-dominated culture of strength. Sofia’s workplace was dominated by men, which meant that the military identity was gender-coded by the dominant group. Anyone who was not of a male gender, or deviated from the given culture in any other way, was therefore at risk of being particularly vulnerable and singled out. Uniformed women’s experiences of being “the other” (i.e., the other gender) in a male-dominated military context where the male eye observes and defines have been clearly illustrated in both international (51, 73–76) and Swedish research (14, 26, 46, 49, 77) in recent decades. There was thus a breeding ground for various types of moral conflict and injury that were related to different views of what was perceived as morally right, proper and desirable from a socialization and identity perspective. Sofia’s range of experiences included frustration, anger and being pissed off, which were strong emotions related to an experience of being let down by superiors who were given legitimate power, in relation to what is perceived as the morally right thing to do in a high-risk situation (4, 5). The morally right thing in Sofia’s case had been a supportive, helpful, respectful and educational cultural context with military generalized others who projected this into a Military Me. Sofia was met with the opposite – hard work and overcompensation were not enough because she was a woman.

#### To suffer from moral injury due to a sudden change of the external frame of meaning: Erika’s case

Erika was an expatriate veteran who was deployed by a government agency other than the Swedish Armed Forces. She had completed many international operations and missions and was in the 25–34 age range. She was still employed by the government agency and on active duty. In line with many other military participants in the study, Erika felt strong emotions when Afghanistan and Kabul fell, but for her the experience was slightly different as she blamed herself for what happened. She was well aware that it was neither rational nor logical. But Erika experienced feelings of disloyalty and betrayal because she was unable to help and avert the course of events.

Erika described it as follows:

I think there is a very strong feeling in me that you have to do the right thing and you have to help those who are struggling, and that I am a capable person who has been trained to do that more than most. […] And because I didn't have the energy or the ability to do that, then yes, this clash between reality and self-image became very heavy to bear. And I felt a lot of guilt and shame about it. I was disappointed in myself and also felt that others were disappointed in me and made others disappointed in me. Yeah, there were a lot of heavy layers to it.

Erika was on sick leave when her mental health was at its worse. In her case, the moral injury occurred in an intense experience of the failure to maintain an identity as a capable and competent service veteran. The failure was reinforced by a strong moral sense expressed in the need to do the right thing and help those who are struggling. When reality in its cruder format met the socially constructed veteran identity, the result was a kind of moral injury caused by a perceived breakdown in character (4, 5, 12). The complexity of Erika’s case embraced both a role as an actor who betrayed, which generated guilt, and a role as a passive witness to events, which created shame. Like Harald, this was neither rational nor objectively logical, but a subjectively experienced moral betrayal and identity collapse that created real and painful suffering.

Erika’s case—and the whole situation of the fall of Kabul—also illustrated how moral injury can occur much later because the conditions of previous actions have radically changed. This changes the entire framework from which a veteran might have based their meaning-making (78). This can raise profound moral questions about perceived failure, betrayal, the meaning of the mission and the purpose of all the suffering and killing.

#### To suffer from moral injury due to leadership betrayal: Roger’s case

Roger was in the 25–34 age range and was a continuously employed soldier and team leader before leaving his employment with the Swedish Armed Forces due to what he perceived as working environment problems and poor leadership. During the international deployment, Roger and his colleagues experienced a problem with a comrade who was not functioning well in their position. A lot was at stake during the international deployment in a high-risk area, for example there was a risk of being injured or killed in an enemy attack. The perceived problem meant that Roger constantly had to stoically compensate for the shortcomings of the comrade and was therefore subjected to considerably more perceived detrimental operational stress than used to be good practice.

Here is what Roger had to say:

Previous missions have always … You rotated the first vehicle, so I was the driver of the first vehicle in my group. And when we're out rolling, we've always rotated between which vehicle drives first, in the group, in the platoon and in the company. But throughout my mission, I was rolling in the front. And we didn't even switch within the group, because another driver was a safety risk. That person was downright unsuitable. So it was an extra stress that I was constantly on … Well, you had to be on full alert. You were looking for objects and enemies everywhere. And I guess that kind of sticks. […] And things just build up. But you have to keep going, so you supress it and don't take care of it. It just keeps building up inside you. Because you can't talk about stuff like that.

Roger and his comrades tried to draw the attention of higher-ups to how this affected their well-being during the deployment, but without any result.

Roger said:

There and then we tried going through the proper channels and said “This person is dangerous. We can't have this person out with us”. We went to our superiors and said “Help us!” and the only response we got was “Figure it out!” […] I don't dwell on it so much today. […] Then I guess you live with the consequences, and it's not right.

Shay’s (4, 5) definition of a moral injury as a perceived betrayal by a legitimate superior in a high-risk situation in relation to what is perceived as morally right was apt in Roger’s experience. He had been struggling with PTSD symptoms as a result of the stress he was subjected to during deployment. Today, a number of years later, Roger was focusing his energy on family, work, trying to feel better and thus leaving behind the perceived betrayal and the experience of poor leadership. Despite this, he expressed bitterness and disappointment in relation to both his superiors and his unit during the interview.

## Discussion

The cases confirm the necessity of all three waves of moral injury as a construct (e.g., a betrayal-based, a transgression-based, and identity or character failure-based moral injury). In addition, the five cases broaden and deepen the understanding of moral injury. Several of the cases suggest that a deep-seated moral injury which emanates from a Veteran *me* may involve lifelong suffering (i.e., an extensive time dimension) even if such moral issues have been addressed in a clinical context. Both Harald and Fanny were “broken” in a sense; to be “broken” as a human being is a profound and taxing existential condition (79–81). The root to and responsibility for this brokenness continues to call for meaningful answers across the whole life span. Such a process can be illustrated as a lifelong existential roller coaster.

The moral injury creates a permanent vulnerability that can feed moral rumination and pain, especially during times in life when resilience is lowered. Moreover, the cases suggest that a military moral injury that emanates from a Veteran *me* may spill over into civilian life and fuel the development of new civilian moral injuries (e.g., the perception of a morally failed identity as a parent or as a partner). Many moral *mes* together constitute the context of the self (40, 41), which means that a moral injury cannot necessarily be isolated from the civilian part of self, identities, and life.

Additionally, a military moral injury may not only consist of a single event, it can involve fundamentally different events and injuries, as in Sofia’s case. Another insight is related to how a moral injury can occur years after returning home without any signs of such impending injury. Each deployment for any Veteran *me* is a high-risk situation with high stakes of various kinds which, in a general sense, creates a potential breeding ground for moral injury. A radically altered frame of meaning can suddenly pull away the ontological rug on which a Veteran *me* stands. This can bring to the fore questions of betrayal in all its forms or other moral issues. All cases suggest that recovery and repair (24) from moral injury are difficult since such an injury can “break” a veteran.

Using a conceptual lens such as moral injury provides the means to better understand and address the different layers of experiences that can cause suffering and negatively impact a person’s mental health (7, 22, 23, 25, 27, 28, 34, 36, 82). Moral injury is not always easy to see and understand, because people are generally not trained to think about their mental health in this way (14). Since the concept has no medical meaning or diagnostical status in a Swedish, or any other, context, it is not something that is regularly screened for or approached in healthcare. There is therefore a potential blind spot, since a moral problem complex is not addressed in a clinical investigation to understand and comprehend why a veteran is struggling with their mental health (26). After all, PTSD represents a relatively narrow clinical lens, and other possible diagnoses may not match a deteriorating mental health condition and its cause either. If a veteran does not meet the criteria, there is a risk that they will end up in a veteran’s health limbo without effective support services and medical care and with more questions than answers. Moral injury has potential as a contemporary concept that complements gaps in a diagnostic conceptual apparatus.

Research and method development for moral injury care has grown over the past decade, especially in North American and Australian contexts (9). Today, many researchers suggest that care and treatment of moral injury can be interdisciplinary, does not have to be organized within a clinical paradigm and context, or even be particularly suitable for cognitive behavioral therapy or other therapeutic approaches (3, 12, 14, 16, 38). This flexibility in approach is necessary because a moral injury is not necessarily an invalid or incorrect mindset of a person that must be reconstructed; it may be a desirable moral implication when seen from a societal perspective.

In other words, society strives to create a moral *me* through values that instill a reluctance to, for example, step over moral thresholds that could create reckless and even dangerous societal members. The generalized other projects morality in a societal *me* that, for example, creates feelings of guilt, shame, disgust, and anxiety in the self if a person makes moral transgressions. Similarly, dejection, bitterness, and anger can ensue if s/he is betrayed by someone else. Groups in society cultivate different types of morality depending on what the group is about to engage and perform. In a military context, there is a military or a warrior culture that is supposed to accept and even embrace the killing of other people (combatants).

This means that it is morally accepted, even, exalted to develop warrior skills and a mindset built around the capacity to kill (50–55). This is both sanctioned by society and fed by the military group, which can put the self in an ambiguous moral field of tension which can affect wellbeing and mental health and increase suffering. Veterans such as Harald can obviously obtain cognitive techniques to broaden the picture and create a wider understanding of an event and its various actors who bear a shared moral responsibility for the death of others. But the root cause of the subjective experience of a moral injury is not addressed in this way because there is no thought error in feeling guilty or ashamed of an act performed or event witnessed. Healing a moral injury goes beyond the reconstruction of an invalid thought structure.

At present, there is an increasing consensus that concepts such as acceptance, forgiveness, reconciliation and vindication are some of the approaches that are central to a process that can alleviate and heal a moral injury (3, 8, 16, 26, 83–85). While it is possible for a moral conflict to be healed, it may be that a moral injury is something the person must reconcile with so that it becomes less painful and more endurable to live with.

Depending on the nature of moral injury and the roles a person may have experienced in such a process—actor, witness, victim—the whole experience can create a complex battery of interrelated moral injuries. Both spiritual approaches [e.g., The Pastoral Narrative Disclosure/PND by (19), and Moral Injury Reconciliation Therapy/MIRT by (20)] and secular ones [e.g., Adaptive Disclosure Therapy/ADT by (17) and Acceptance and Commitment Therapy/ACT described by clinical researchers such (86, 87)] can gravitate toward concepts such as acceptance, forgiveness, reconciliation, and vindication of a moral injury. However, these are concepts that have their origins in ancient spiritual and religious wisdom practices and traditions (2). This can be a challenge from a Swedish healthcare perspective, among other things because Sweden is often described as one of the world’s most secular countries [see (88, 89)].

At the same time, Sweden is highly diverse and from an international point of view quite unique in regards to its military spiritual care. Military spiritual care has persisted through unbroken traditions within the Swedish Armed Forces since the 1500s, that is, when Sweden as a nation began to be organized (90). Moral injury may be a new concept, but the phenomenon itself is old and timeless. While researchers, clinicians and practitioners in a Swedish context have quite recently begun to understand moral injury, there is another group in practice since the 1500s that has taken on and cared for the existential challenges among military personnel and veterans.

Military chaplains do not necessarily master and apply the entire conceptual apparatus of moral injury, but draw and operate from a soon-to-be 500-year-old wisdom tradition around military spiritual care that gravitates toward the life issues and existential concerns of military personnel and veterans during conscription, military training, war zone deployments, and in the aftermath of deployments (91). Military chaplains are existential experts in which ethics and moral injury are included. They constitute the foremost group of existential practitioners that are systematically represented in the Swedish Armed Forces in general, amid deployment, and in the veteran context. Many of the military chaplains are also deployment veterans, which gives them a unique cultural competence and understanding of war zone deployments (26, 90).

Even in the United States, where research into the treatment of moral injury has the longest history (9, 32), there is as yet no gold standard. Yet such a standardization may not be either achievable or desirable, given that morality is an expression of highly diverse communities and societies, and that moral injury may occur at the intersection of a wide variety of roles and events. These complexities may instead suggest the need for many approaches, with selection determined by context, identities, and veteran preferences. If events, different roles during one or several events, various military or other organizations and war zone contexts, as well as societal contexts are integrated then this manifests as extremely complex, even nebulous. Rather, these many intricacies suggest manifold possible approaches or methods which can be employed depending on what resonates in the veteran considering the context, identities and preference (3, 9, 16, 26, 84).

### Limitations and directions for future research

There are several limitations to this study, which should be taken as directives for future research. First, while this qualitative approach allowed for a deeper exploration of subjective experiences, these experiences are unique to the participants themselves and not necessarily representative of the veteran population(s) in Sweden and elsewhere. However, given that the narrative accounts yielded themes that were applicable to all (e.g., moral conflicts and injuries, saliency of veteran identity) suggest commonalities that extends beyond this interview sample. Future research should examine the applicability and utility of these moral injury themes in the narratives of other military veterans who struggles with deteriorating mental health without fulfillment of clinical PTSD. In addition, the qualitative results provide several domains that can inform quantitative measurement development encompassing several subscales that include moral injury and identity.

The results also suggest that moral conflicts and injuries can be related to the participants’ time in the military as well as to events outside of that timeframe (i.e., civilian life). Hence, in healthcare settings a wider focus is suggested that accounts for the continuity of experiences and fluidity of identities across the military and civilian spheres to fully address struggles due to a time dimension. Veterans grappling with moral issues are engaged in a longer cognitive and emotional rumination and reflection process. The meaning making involved in the moral issues of life in general, may require addressing specific moral and spiritual failures that occurred in various areas (e.g., military, civilian) over one’s lifetime (e.g., past, present). With a growing moral awareness there is also a greater sensitivity to such issues. As such, screening for moral conflicts and injuries [e.g., (25, 29, 31, 32)] among veterans in the health care setting should not only be a golden standard, it should also be conducted among service members after deployment as part of prevention and early intervention efforts (78).

## Data availability statement

The original contributions presented in the study are included in the article/supplementary material, further inquiries can be directed to the corresponding author.

## Ethics statement

The studies involving humans were approved by The Swedish Ethical Review Authority with the reference number 2021-05410-01. The studies were conducted in accordance with the local legislation and institutional requirements. The participants provided their written informed consent to participate in this study. Written informed consent was obtained from the individual(s) for the publication of any potentially identifiable images or data included in this article.

## Author contributions

The author confirms being the sole contributor of this work and has approved it for publication.

## Footnotes

^1^The Veterans Clinic caters for people who have served overseas in a war zone or disaster area on behalf of the Swedish Government, for assessment and treatment of PTSD or other mental illness related to this service. The veterans who are either referred to the Veterans Clinic or request care themselves come there because they feel unwell. The clinic does important work for veterans in Sweden, with more than 300 patients admitted since it opened its doors. Of this patient flow, half meet the criteria for clinical PTSD, while the other half do not meet the criteria despite experiencing a marked deterioration in mental health and increased suffering (14).
